# Natriuretic Peptides as Biomarkers for Congestive States: The Cardiorenal Divergence

**DOI:** 10.1155/2017/1454986

**Published:** 2017-06-18

**Authors:** Abhilash Koratala, Amir Kazory

**Affiliations:** Division of Nephrology, Hypertension, and Renal Transplantation, University of Florida, Gainesville, FL, USA

## Abstract

Congestion represents the primary reason for hospitalization of patients with heart failure and is associated with adverse outcomes. Fluid overload has been shown to be inadequately addressed in a significant subset of these patients in part due to lack of robust, reliable, and readily available biomarkers for objective assessment and monitoring of therapy. Natriuretic peptides have long been used in this setting, often in conjunction with other assessment tools such as imaging studies. Patients presenting with concomitant cardiac and renal dysfunction represent a unique population with regard to congestion in that the interactions between the heart and the kidney can affect the utility and performance of biomarkers of fluid overload. Herein, we provide an overview of the currently available evidence on the utility of natriuretic peptides in these patients and discuss the clinical conundrum associated with their use in the setting of renal dysfunction. We highlight the potential divergence in the role of natriuretic peptides for assessment of volume status in a subset of patients with renal dysfunction who receive renal replacement therapy and call for future research to elucidate the utility of the biomarkers in this setting.

## 1. Background

Heart failure (HF) is a major public health problem because of its high prevalence, poor prognosis, and healthcare cost burden. The prevalence of HF in adults over 20 years of age in the United States was estimated to be 2.4% in 2008, and by 2030, an additional 3 million people are predicted to develop HF, which is a 25% increase in prevalence from 2010 [1]. Congestion is recognized as the major cause for hospitalization in the vast majority of patients with HF and contributes to adverse outcomes [2]. Nevertheless, a significant proportion of the patients admitted to the hospital for acute decompensated heart failure (ADHF) is discharged with unresolved congestion. A report of more than 50,000 patients in the ADHF National Registry revealed that about 33% of the patients lose as little as less than 2.3 kg and another 16% even gain weight during the course of hospitalization [3]. Congestion often remains unrecognized until conditions develop that warrant hospital admission. Elevated left ventricular filling pressures are present in a significant subset of HF patients with no obvious clinical signs; termed “hemodynamic congestion” in contrast to “clinical congestion” that constitutes constellation of signs and symptoms including shortness of breath, orthopnea, pulmonary rales, peripheral edema, and jugular venous distention [4]. In addition, congestion is one of the contributing factors for worsening renal function (WRF) in the setting of ADHF, which in turn is thought to adversely affect outcomes. Interestingly, WRF can also occur while patients are being treated for congestion especially with diuretic therapy. With recognition of the importance of early detection of congestion, there has been renewed interest in investigating novel circulating serum and plasma biomarkers in patients with HF. An ideal biomarker should have the following three characteristics to be clinically useful. Firstly, it should be accurate with reasonable cost and short turnaround times; secondly, it should provide additional information that is not obtainable from a thorough clinical assessment; and finally, its measurement should aid in clinical decision making [5, 6]. The role of various biomarkers has been studied in diagnosing, grading the severity, and predicting the progression of HF as an adjunct to clinical parameters and invasive testing. B-type natriuretic peptide (BNP) and N-terminal prohormone of BNP (NT-proBNP), which are a part of natriuretic peptide system, are frequently used in the clinical practice for this purpose. In this article, we briefly discuss the utility and performance characteristics of these biomarkers in the setting of HF and discuss the impact of concomitant renal dysfunction on its application in this setting.

## 2. The Natriuretic Peptide System

Atrial natriuretic peptide (ANP), BNP, and C-type natriuretic peptide (CNP) constitute the human natriuretic-peptide family. Among these, ANP was the first to be discovered in the 1980s. It is a 28-amino acid polypeptide resulting from the C-terminal end of the prohormone proANP and secreted mainly by the atria. BNP was initially isolated from brain tissue, but is also found in the circulation, and the highest concentration is found in the cardiac ventricles. Prior to its activation, BNP is stored as a 108-amino acid polypeptide precursor, proBNP, in both cardiac ventricles and, to a lesser extent, in the atria. ProBNP is cleaved in response to volume expansion and myocyte stretch to produce the biologically active 32-amino acid BNP and the 76-amino acid peptide, NT-proBNP. CNP is primarily found in the brain, and plasma concentrations are typically low. Despite its name, CNP does not possess natriuretic effect but does have vasodilatory properties and can be synthesized by vascular endothelial cells [7, 8]. Plasma ANP and BNP concentrations increase in response to volume overload and pressure overload in the heart and are considered as physiological antagonists for the effects of angiotensin II on vascular tone, aldosterone secretion, renal-tubular sodium reabsorption, and vascular-cell growth, thereby producing diuretic, natriuretic, and antihypertensive effects [7, 9]. On the other hand, plasma CNP concentrations change very little with cardiac overload but this peptide likely has paracrine role in the regulation of vascular tone [9]. Natriuretic peptide receptor type C and neutral endopeptidases actively clear BNP from the circulation in addition to renal clearance; the plasma half-life is thus short, approximately 20 minutes. On the other hand, NT-proBNP is primarily cleared by renal excretion and has a relatively prolonged half-life of approximately 120 minutes. This is the likely explanation for higher serum values of NT-proBNP, which is approximately 6 times higher than BNP values, though both these molecules are produced in equal proportions [10]. Although ANP was identified first, concentrations of BNP in the myocardial tissue were found to be higher than those of ANP. So, BNP has been studied more intensely than ANP as a biomarker of increased ventricular filling pressure and left ventricular dysfunction.

## 3. Natriuretic Peptides and Heart Failure

### 3.1. Diagnosis

It is well known that BNP and NT-proBNP play an important role in the diagnosis of patients presenting with dyspnea of uncertain cause [11, 12]. In the prospective study better known as “Breathing Not Properly Multinational Study,” Maisel et al. evaluated 1586 patients who came to the emergency department with acute dyspnea and measured BNP with a bedside assay [11]. The final diagnosis was HF in 47 percent (confirmed by chest radiograph and/or echocardiography and other clinical tests), no HF in 49 percent, and noncardiac dyspnea in patients with a past history of left ventricular dysfunction in 5 percent. At levels of less than 50 pg/ml, BNP had a negative predictive value of 96 percent suggesting that it can be a good “rule out” test in the acute setting [11].

### 3.2. Guidance of Therapy

Biomarker-guided therapy has been shown to portend favorable outcomes in the setting of HF. For example, in a randomized controlled multicenter Trial of Intensified versus Standard Medical Therapy in Elderly Patients with Congestive Heart Failure (TIME-CHF) including 499 patients, the investigators sought to know whether intensified HF therapy guided by N-terminal BNP is superior to a symptom-guided therapy [13]. After an 18-month follow-up period, improvement in patients' quality-of-life metrics was similar in both groups. However, compared with the symptom-guided group, survival free of hospitalization for HF was significantly higher among those in the N-terminal BNP-guided group (72% versus 62%, resp.; hazard ratio 0.68; *P* = 0.01). Interestingly, HF therapy guided by N-terminal BNP improved outcomes in patients aged 60 to 75 years but not in those aged 75 years or older (*P* < 0.02) [13].

In line with the above findings, a meta-analysis of 6 randomized controlled trials with a total of 1627 patients has found that intensification of medical therapy tended to be significantly greater with biomarker guidance with regard to the use of angiotensin converting enzyme inhibitors or angiotensin receptor blockers, *β*-blockers, and aldosterone receptor antagonists, the 3 classes of drugs that have been definitively shown to reduce mortality in chronic HF [14]. A large randomized controlled trial known as GUIDE-IT (Guiding Evidence Based Therapy Using Biomarker Intensified Treatment in Heart Failure) is currently underway and is expected to provide more insights into natriuretic peptide-guided therapy in high-risk patients with systolic HF [15].

### 3.3. Prognosis

It has been shown that changes in natriuretic peptide levels over time in response to therapy are powerful indicators of prognosis, that is, patients in whom natriuretic peptide levels decrease in response to ongoing medical management seem to have a better prognosis than those with increased or similar levels compared to that of initial presentation. For instance, in a study by Latini et al., 3740 patients were divided into 4 groups according to their baseline BNP levels versus 4 months or 12 months later, labelled as low → low, high → high, high → low, and low → high [16]. Patients who improved their BNP levels at 4 months (high → low) were found to have a similar risk for mortality compared with the low → low group (hazard ratio = 1.191, *P* = 0.2746). On the other hand, patients in whom the BNP levels increased (low → high) had a higher risk of mortality compared to those in the low → low group (hazard ratio 2.578, *P* < 0.0001) and were indistinguishable from the high → high group. Worsening of BNP (low → high) was associated with 0.03 cm/m^2^ increase in left ventricular end-diastolic diameter while it decreased by 0.10 cm/m^2^ in high → low and low → low groups (*P* < 0.001) [16].

Also, in a study on 1742 patients with heart failure, Masson et al. compared the prognostic discrimination of a single determination of NT-proBNP (baseline or 4 months) versus continuous changes, expressed as either relative (percent) or absolute changes [17]. They found that a single determination of NT-proBNP showed a higher prognostic discrimination than continuous changes of concentrations, expressed either as absolute or relative changes (area under the curve at 4 months: 0.702, 95% confidence interval 0.669 to 0.735).

## 4. Natriuretic Peptides and Renal Dysfunction

**Figure 1:**
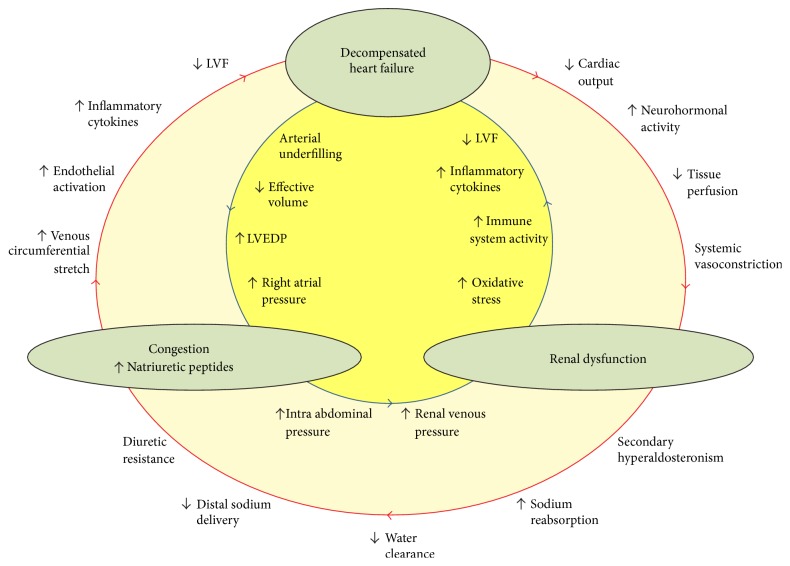
Bidirectional pathways linking heart failure, renal dysfunction, and congestion in cardiorenal syndrome. Decompensation of heart failure can lead to deterioration in renal function via exacerbated neurohormonal activity (i.e., low forward flow) or through fluid overload and renal venous congestion (i.e., high backward pressure). Increase in natriuretic peptides represents the congestive state in this setting. Adapted with permission from reference [27]. LVEDP: left ventricular end-diastolic pressure; LVF: left ventricular function.

HF is commonly associated with various degrees of renal dysfunction. Indeed, it is estimated that over 50% of patients with a diagnosis of ADHF develop decrease in glomerular filtration rate (GFR) [18]. The interplay between cardiac and renal dysfunction, also known as the “cardio-renal interaction,” is mediated through a cascade of complex mechanisms and is associated with a significant increase in the risk of morbidity and mortality (Figure 1) [19].

HF patients with renal impairment have higher concentrations of BNP and NT-proBNP. As mentioned above, NT-proBNP was thought to be more dependent on renal excretion compared to BNP as the latter is also cleared from circulation by natriuretic peptide receptor type C and neutral endopeptidases. However, the recent data does not support this notion. It has been shown that the kidneys clear both these hormones equally, thus potentially increasing their concentrations in the setting of renal impairment. In a study of 165 subjects, van Kimmenade et al. combined measurements of BNP and NT-proBNP levels in the renal arteries and veins via renal arteriography with invasive renal plasma flow measurements and echocardiography and calculated fractional extraction (FE) of these peptides [20]. The BNP and NT-proBNP concentrations correlated similarly to GFR (*r* = −0.35 and *r* = −0.30, resp.; *P* < 0.001 for both), but the NT-proBNP/BNP serum ratio was negatively associated with GFR (*r* = −0.21, *P* = 0.008). Although FE (BNP) and FE (NT-proBNP) correlated strongly with each other (left: *r* = 0.66; right: *r* = 0.60; *P* < 0.001 for both), the left and right FE (NT-proBNP/BNP) ratios were not impacted by GFR (*r* = 0.10, *P* = 0.30 and *r* = 0.08, *P* = 0.43, resp.). It is of note that most subjects had a GFR ≥30 ml/min/1.73 m^2^ in this study, and the study population consisted of hypertensive patients [20].

Given the increased levels of natriuretic peptides in patients with renal impairment, a number of studies have tried to elucidate their utility in this setting. In a study of 599 dyspneic patients with GFR ranging between 15 and 252 ml/min/1.73 m^2^, Anwaruddin et al. examined the interaction between renal function and NT-proBNP levels [19]. They found that worse renal function was associated with cardiac structural and functional abnormalities on echocardiography; NT-proBNP and GFR were inversely and independently related (*P* < 0.001), and NT-proBNP values > 450 pg/ml for patients ages < 50 years (>900 pg/ml for patients ≥ 50 years) had a sensitivity of 85% and a specificity of 88% for diagnosing ADHF among subjects with GFR ≥ 60 ml/min/1.73 m^2^. Using a cut-point of 1200 pg/ml for subjects with GFR < 60 ml/min/1.73 m^2^, they found sensitivity and specificity to be 89% and 72%, respectively. The investigators concluded that NT-proBNP testing, using appropriate cutoffs, is valuable for the evaluation of the dyspneic patients with suspected HF irrespective of renal function [19]. Larger prospective and randomized controlled trials are needed to further evaluate these findings.

In addition to surrogate end points such as abnormal cardiac structural and functional findings, BNP and NT-pro-BNP are also associated with hard outcomes in patients with chronic kidney disease (CKD). For instance, in a Chinese study that included 999 patients with coronary artery disease, it was found that the crude and multiple adjusted hazard ratios of NT-proBNP to detect HF and predict mortality were significantly higher in patients with CKD compared with the remainder of the cohort [21]. In that study, NT-proBNP detected HF with a cutoff value of 298.4 pg/ml in non-CKD patients and a cutoff value of 435.7 pg/ml in CKD patients. NT-proBNP predicted death with a cutoff value of 369.5 pg/ml in non-CKD patients and a cutoff value of 2584.1 pg/ml in CKD patients. Similarly, the African American Study of Kidney Disease and Hypertension trial including hypertensive blacks with a GFR of 20 to 65 ml/min/1.73 m^2^ and no other identified cause of kidney disease found that patients with elevated NT-pro-BNP had a 4 times higher hazard for cardiovascular events than those with undetectable levels [22]. This association not only applies to the general CKD population, but also to renal transplant recipients. It is known that HF is prevalent after transplantation and is associated with poor prognosis [23, 24]. In a study including 606 renal transplant recipients compared with a general population cohort of 3234 subjects, Oterdoom et al. found that association of NT-proBNP with mortality was significantly steeper in the transplant cohort compared with the general population [25]. Risk for mortality was similar for transplant recipients and general population with low NT-proBNP levels (<100 pg/ml).

Finally, in a meta-analysis Schaub et al. recently pooled together the studies on BNP who had a subgroup analysis for those patients with renal dysfunction [26]. This study included a total of 4287 patients and was designed to answer 2 important questions: whether renal dysfunction alters the diagnostic ability of NT-proBNP to detect ADHF and whether renal dysfunction alters the prognostic ability of NT-proBNP. It was found that the correlation coefficients between estimated GFR and NT-proBNP were statistically significant and ranged from −0.21 to −0.58, meaning NT-proBNP levels consistently increase as renal function declines. With regard to the diagnostic ability of NT-proBNP, the cut-points in patients with an estimated GFR < 60 ml/min/1.73 m^2^ were roughly two-fold higher than the cut-points in patients with an estimated GFR > 60 ml/min/1.73 m^2^, but even with higher cut-points, the specificity and sensitivity were only slightly lower in patients with estimated GFR < 60 compared to those with estimated GFR > 60. In terms of the prognostic ability of NT-proBNP, the pooled risk ratios between patients with preserved and diminished renal function were not significantly different (*P* = 0.652) although there was a higher event rate in patients with preserved renal function compared to those with renal dysfunction. The result of this study suggests that markedly higher concentrations of NT-proBNP in a patient with renal dysfunction may be partially due to decreased clearance, but it still portends a higher absolute risk for mortality compared to patients with normal renal function [26].

Ultrafiltration therapy represents an alternative to conventional diuretic-based regimens for management of congestion and fluid overload in patients with ADHF [27]. In this novel, therapeutic strategy fluid is mechanically extracted from plasma in the extracorporeal circuit and hemofilter. So far, studies evaluating the role of ultrafiltration therapy in ADHF have largely used clinical assessment such as weight reduction and congestion-related symptoms to guide their therapy [28]. Measurement of weight is prone to errors in critically ill patients (e.g., due to fluid redistribution rather than accumulation in a subset of patients with ADHF), and the role of bioimpedance is not clear due to a significant number of these patients having associated renal dysfunction. As such, assessment of congestion is largely subjective. Future studies are needed to evaluate the potential role of serial measurements of plasma BNP levels for objective monitoring of volume status in these patients during therapy for effective and safe extraction of excess fluid.  Table 1 summarizes selected ultrafiltration studies that reported the changes in natriuretic peptide levels and renal function associated with decongestion.

## 5. Natriuretic Peptides and Renal Replacement Therapy

The concentrations of natriuretic peptides are elevated in patients on dialysis. As the likely stimulus for their release is volume overload, ANP and BNP have been investigated as potential markers of volume status in this patient population. Earlier studies reported a low clearance for natriuretic peptide fragments [36, 37]. Moreover, clinical studies have shown that ANP levels decrease when dialysis is associated with fluid removal, but not when dialysis is performed without extraction of fluid [38]. This supports the notion that changes in serum levels of natriuretic peptides are mainly due to decongestion rather than clearance through dialysis.

BNP outperforms ANP in the prediction of left ventricular hypertrophy and dysfunction [39], and cohort studies in the dialysis population have demonstrated a direct association between NT-proBNP levels and the risk of cardiovascular and all-cause mortality [40–42]. For instance, in a study including 2990 incident hemodialysis patients, increasing quartiles of NT-proBNP were associated with a monotonic increase in 90 days (hazard ratio (HR) 1.7–6.3, *P* < 0.001) and 1 year (HR 1.7–4.9, *P* < 0.001) all-cause mortality. Also, patients with the greatest increase in NT-proBNP after 3 months of dialysis had a 2.4-fold higher risk of mortality compared to those with the greatest decrease in NT-proBNP [42].

The role of routine serial BNP and NT-proBNP testing in dialysis patients to monitor the cardiovascular risk is not clear. Also, between-person and within-person variations in the concentrations of biomarkers have to be taken into account before making such determination. In a prospective cohort study in 55 prevalent and hemodialysis patients, Fahim et al. performed serial assessments including NT-proBNP testing, clinical review, electrocardiography, and bioimpedance spectroscopy [43]. Respective between- and within-person coefficients of variation were 153% and 27% for weekly measurements, and 148% and 35% for monthly measurements suggesting that the between-person variation of NT-proBNP was significantly greater than within-person variation and NT-proBNP testing might better be applied in the dialysis patients using a relative-change strategy. Interestingly, Within-person variation was not affected by dialysis modality, inflammation, hydration status, or cardiac comorbidities [43].

Determination of intravascular volume is an integral part of adequate dialysis prescription and there was hope for using natriuretic peptides for objective assessment of volume status in this setting. Unfortunately, the data so far has been conflicting. For example, in a study of 39 patients undergoing hemodialysis thrice weekly, pertinent data was collected at the start and end of each of 3 consecutive hemodialysis sessions [44]. Pre- and postdialysis plasma BNP levels, blood pressure, and weight were considered. Investigators found no correlation between changes in intradialytic BNP values and other measured parameters. Plasma volume changes were measured in a subset of 13 patients and showed minimal change during dialysis. Similarly, a prospective study on 51 stable peritoneal dialysis patients did not show a significant correlation between clinical assessment of volume status and BNP concentrations (*P* = 0.76) [45]. Interestingly, there was also no correlation between volume status and thoracic fluid content measured by bioimpedance in that study (*P* = 0.39). One of the criticisms of these studies is that they examined stable patients with relatively lower BNP levels with values not higher than 500 pg/ml. In contrast, in an observational study including 19 consecutive dialysis patients hospitalized for various indications with a mean baseline ejection fraction of 43.8%, Tapolyai et al. proposed the validity of BNP-directed ultrafiltration [46]. In this study, all patients were hypervolemic at admission according to BNP criteria of >500 pg/ml (mean 2412 ± 1479 pg/ml) and 42% were identified to have HF based on clinical criteria. This means hypervolemia was clinically appreciable in less than half of the patients. Patients were ultrafiltered daily until they achieved a target BNP level of less than 500 pg/ml (maximum 5 l per session). At the end of the study, the mean BNP was significantly reduced from 2412 to 1245 pg/ml (*P* = 0.0013), body weight was reduced by a mean of 11 kg (*P* = 0.0002), systolic blood pressure decreased by 22 mmHg, and diastolic blood pressure by 12 mmHg (*P* = 0.0222 and 0.0139, resp.) [46].

Therefore, the currently available data suggests that the performance characteristics and utility of natriuretic peptides for determination of congestion and volume status in the dialysis population are not similar to patient with HF and fluid overload. Larger randomized controlled trials are needed to confirm these findings and evaluate the role of BNP as a biomarker in routine practice for patients receiving maintenance dialysis.

## 6. Inhibiting Degradation of Natriuretic Peptides: Clinical Implications

Neprilysin is a widely expressed neutral endopeptidase that degrades all three members of the natriuretic family including ANP, BNP, and CNP, but not NT-proBNP [47]. Recently, LCZ696, a complex of the neprilysin inhibitor sacubitril and the angiotensin receptor blocker valsartan was approved for the treatment of HF with reduced ejection fraction. As neprilysin is thought to be responsible for degrading BNP, it is expected that patients who are treated with LCZ696 will have higher plasma BNP levels due to the inhibition of neprilysin activity. On the other hand, NT-proBNP levels are not expected to be influenced by neprilysin inhibition. Supporting this assumption, a recent study comparing LCZ696 with the angiotensin converting enzyme inhibitor enalapril in 8399 patients with HF and reduced ejection fraction has shown that levels of plasma BNP were higher during treatment with LCZ696 than with enalapril, but circulating levels of NT-proBNP were lower (*P* < 0.0001, at 8 months) [48]. Consequently, it is reasonable to assume that NT-proBNP might be a better marker to follow the therapy than BNP in patients being treated with this medication. However, on a broader note, the utility of natriuretic peptides in the context of neprilysin inhibition is limited by a number of factors. First, the beneficial effect of LCZ696 in patients with preserved ejection fraction or patients with acute HF has not been established. In addition, it is unknown whether neprilysin is capable of degrading intact proBNP and if measurement of proBNP can be of use in these patients. Moreover, with the recent data showing that elevated BNP levels might inhibit the activity of circulating neprilysin, the interpretation becomes even more difficult [49]. Considering the complexity of the natriuretic peptide system and the diversity of HF states, the question remains as to whether BNP or NT-proBNP alone should be used in order to fully understand the clinical status of these patients and determine appropriate management strategy.

## 7. Other Biomarkers for Heart Failure

While natriuretic peptides are the most rigorously evaluated biomarkers in HF, there are several other markers that have potential to aid in clinical decision making and seem to be promising targets. Moreover, combining more markers might help in better characterization of patients with HF and thereby create newer options for treatment and identification of patients that need a closer follow-up, such as those with concomitant renal dysfunction.

HF biomarkers can be broadly grouped into markers of inflammation (e.g., C-reactive protein, myeloperoxidase), markers of fibrosis and extracellular remodeling (e.g., procollagen, galectin-3, ST2), markers of mechanical strain (e.g., natriuretic peptides), markers of hemodynamic homeostasis (e.g., copeptin, adrenomedullin), and markers of cardiomyocyte injury (e.g., troponins) [6, 50]. Although the detailed description of these biomarkers is beyond the scope of this review, a brief overview of selected ones follows.

C-reactive protein (CRP) is a pentameric protein and a well-known marker of inflammation. Elevated levels of CRP have been observed in HF patients, especially in acute exacerbations [51]. To investigate the utility of CRP as a biomarker in HF, Alonso-Martínez et al. studied 76 patients with HF and mean CRP level of 3.94 mg/dL admitted to the hospital, independent of the cause [52]. While the mean left ventricular ejection fraction was 50.41, the investigators observed a trend of higher CRP levels in relation to ejection fractions below 35%. In addition, CRP levels on discharge increased in relation to the New York Heart Association (NYHA) HF class: I: 0.74 ± 0.69; II: 3.78 ± 3.76; III: 7.4 ± 8.65; IV: 12.2 ± 15.27 (*P* < 0.05). Also, among the patients who were readmitted during the course of the 18-month follow-up, those presenting CRP levels > 0.9 mg/dL were identified as candidates for earlier hospitalization than those with levels < 0.9 mg/dL (*P* = 0.02). The authors concluded that higher CRP levels are associated with higher functional class and could be an independent marker of improvement and readmission in HF [52]. However, CRP was not shown to have prognostic significance in other studies [53, 54]. Moreover, studies did not demonstrate decrease in CRP levels in response to the common HF treatments, namely angiotensin converting enzyme inhibitors and spironolactone [55, 56]. Therefore, in spite of being readily available, CRP measurement is not routinely used as a diagnostic or prognostic tool in HF.

ST2 is a member of the toll-like/interleukin-1 receptor superfamily, and its expression is known to be induced by a mechanical strain in cardiac myocytes [57]. In a study on 593 patients presenting with ADHF, it was shown that ST2 concentrations are strongly predictive of mortality at 1 year [58]. Also, elevated ST2 concentrations were shown to predict sudden cardiac death in patients with chronic HF and provide complementary information to NT-proBNP levels [59]. Galectin-3 is a *β*-galactosidase binding lectin expressed and secreted by activated macrophages. Several studies have been performed evaluating the diagnostic and prognostic potential of galectin-3 in HF with conflicting results. A recent meta-analysis of 27 articles found that galectin-3 was ineffective in predicting all-cause and cardiovascular mortalities in HF patients [60]. However, it is notable that the combination of natriuretic peptides and galectin-3 could be superior in predicting mortality compared to either of the biomarkers alone [60].

Cardiac troponins T and I, the markers of myocyte injury, have emerged as sensitive and specific markers of myocyte injury in the recent past and have improved the diagnosis, risk stratification, and care of patients with acute coronary syndromes. It has been shown that cardiac troponin I is detected in 25–33% patients with severe HF and is a powerful predictor of mortality at 3 months [61]. Similarly, in a prospective study including 136 ambulatory patients with HF, it was found that those with elevated troponin T were at increased risk of death or hospitalization (RR 2.7, 95% CI 1.7–4.3, *P* = 0.001) and death alone (RR 4.2, 95% CI 1.8–9.5, *P* = 0.001) [62]. Other myocardial proteins such as myosin light chain 1, heart fatty-acid binding protein, and creatine kinase MB fraction are also found in stable patients with severe HF. Similar to cardiac troponins, the presence of these myocardial proteins in the serum is a predictor of death or hospitalization for HF [63].

## 8. Conclusion

The natriuretic peptide testing is an established tool in diagnosing, prognosticating, and guiding treatment of patients with HF. Limitations for their use do exist such as in all stages of renal impairment. Nevertheless, it remains a useful test in this patient population. Normal plasma BNP level has a high negative predictive value, effectively excluding the presence of HF in both dialysis and nondialysis CKD patients and possibly eliminating the need for additional expensive testing. NT-proBNP appears to perform similarly to BNP in patients with renal dysfunction and is subject to similar limitations. However, in patients receiving hemodialysis, these biomarkers show suboptimal performance assessment of congestion and volume status and cannot be used to guide fluid removal in this setting. With many novel biomarkers on the horizon, future research should focus on investigating multimarker strategy, similar to the setting of acute kidney injury (i.e., use of a biomarker panel) for complex patients with HF. Moreover, it would be of interest to study whether ultrafiltration therapy in ADHF can be guided by these biomarkers for effective and safe extraction of excess fluid.

## Figures and Tables

**Table 1:** Changes in natriuretic peptides in patients treated for acute heart failure.

First author (year)	Number of UF patients	Age (years)	Male gender (%)	Decrease in weight (Kg)	Fluid removed (liters)	Change in BNP (pg/ml)	Change in Scr (mg/dL)
Costanzo (2005) [29]	20	74.5	75	6	8.65	−442 at discharge	No significant change (+0.08 at discharge)
Costanzo (2007) [30]	100	62	70	5	4.6	NA (baseline 1256; similarly improved in both groups)	No significant change (+0.3 at 72 hours)
Giglioli (2011) [31]	15	72.4	87	5.43	9.3	−3266 at 36 hours (NT-proBNP)	No significant change (−0.55) at 36 hours
Hanna (2012) [32]	19	60	84.2	4.7	5.2	−2291 at 48 hours (NT-proBNP)	No significant change (+0.2) at 48 hours
Bart (2012) [33]	94	69 (median)	78	5.7	7.44	−814 at 96 hours (NT-proBNP)	+0.23 at 96 hours
Jefferies (2013) [34]	87 (HFLEF) 97(HFPEF)	65 (HFLEF) 67 (HFPEF)	64 (HFLEF) 46 (HFPEF)	7.57 (HFLEF) 6.39 (HFPEF)	11.14 (HFLEF) 10.6 (HFPEF)	−211 (HFLEF) −88 (HFPEF) at discharge	+0.22 (HFLEF) no significant change in HFPEF group (+0.9) at discharge
Costanzo (2016) [35]	110	67	69.1	10.7 at 72 hours	18.7	−250 at discharge	+0.12 at discharge

+ and − before a number indicate “increase by” and “decrease by,” respectively. Scr: serum creatinine; NA: not available; HFLEF: heart failure with low ejection fraction; HFPEF: heart failure with preserved ejection fraction; UF: ultrafiltration.

## Abbreviations

HF: Heart failure
ADHF: Acute decompensated heart failure
WRF: Worsening renal function
BNP: B-type natriuretic peptide or brain natriuretic peptide
NT-proBNP: N-terminal prohormone of BNP
ANP: Atrial natriuretic peptide
CNP: C-type natriuretic peptide
NYHA: New York Heart Association
GFR: Glomerular filtration rate
FE: Fractional extraction
CKD: Chronic kidney disease
HR: Hazard ratio
CRP: C-reactive protein
RR: Relative risk.
